# N-terminal phosphorylation of xHes1 controls inhibition of primary neurogenesis in *Xenopus*

**DOI:** 10.1016/j.bbrc.2018.12.135

**Published:** 2019-02-05

**Authors:** Laura J.A. Hardwick, Anna Philpott

**Affiliations:** a Department of Oncology, University of Cambridge, Hutchison/MRC Research Centre, Cambridge Biomedical Campus, Cambridge, CB2 0XZ, UK; b Wellcome Trust/MRC Cambridge Stem Cell Institute, University of Cambridge, Tennis Court Road, Cambridge, CB2 1QR, UK; c Peterhouse, University of Cambridge, Trumpington Street, Cambridge, CB2 1RD, UK

**Keywords:** xHes1, bHLH, Neurogenesis, *Xenopus*, Phosphorylation, Neurogenin2, bHLH, basic-Helix-Loop-Helix, Cdk, Cyclin-dependent-kinase, ISH, In situ hybridisation, SP, Serine-Proline, TP, Threonine-Proline, WT, Wild-Type

## Abstract

The processes of cell proliferation and differentiation are intimately linked during embryogenesis, and the superfamily of (basic) Helix-Loop-Helix (bHLH) transcription factors play critical roles in these events. For example, neuronal differentiation is promoted by class II bHLH proneural proteins such as Ngn2 and Ascl1, while class VI Hes proteins act to restrain differentiation and promote progenitor maintenance. We have previously described multi-site phosphorylation as a key regulator of tissue specific class II bHLH proteins in all three embryonic germ layers, and this enables coordination of differentiation with the cell cycle. Hes1 homologues also show analogous conserved proline directed kinase sites. Here we have used formation of *Xenopus* primary neurons to investigate the effects of xHes1 multi-site phosphorylation on both endogenous and ectopic proneural protein-induced neurogenesis. We find that xHes1 is phosphorylated *in vivo*, and preventing phosphorylation on three conserved SP/TP sites in the N terminus of the protein enhances xHes1 protein stability and repressor activity. Mechanistically, compared to wild-type xHes1, phospho-mutant xHes1 exhibits greater repression of Ngn2 transcription as well as producing a greater reduction in Ngn2 protein stability and chromatin binding. We propose that cell cycle dependent phosphorylation of class VI Hes proteins may act alongside similar regulation of class II bHLH proneural proteins to co-ordinate their activity.

## Highlights

• xHes1 is phosphorylated in *Xenopus* embryos on conserved N terminal SP/TP sites.
• *In vitro*, xHes1 protein can be phosphorylated by Cyclin-dependent-kinases.
• Under-phosphorylated xHes1 has increased protein stability relative to WT xHes1.
• Under-phosphorylated xHes1 has enhanced inhibitory activity during neurogenesis.
• xHes1 reduces Ngn2 expression, protein stability and chromatin binding.

## 1 Introduction

Throughout embryogenesis, cell proliferation, fate choice and differentiation must be closely coordinated in time and space; in many tissues, the evolutionarily conserved superfamily of helix-loop-helix (HLH) transcription factors play central roles in all three of these processes [1]. This superfamily is defined by a conserved HLH motif that mediates protein dimerisation, and additionally, most members contain a DNA binding basic domain (bHLH proteins). However, outside of this region, significant diversity of sequence, structure and function allows identification of distinct sub-groups within the superfamily with differing and sometimes opposing regulatory roles [2].

For example, early studies on *Drosophila* neurogenesis established a model for selection of neuronal precursors from otherwise equivalent neuroectodermal cells, based on antagonistic interaction between tissue specific class II activating bHLH proneural proteins such as *achaete-scute complex* and *atonal*, and the inhibitory bHLH-Orange class VI proteins of the *hairy-and-enhancer-of-split* family [3]. Within the mammalian nervous system, the class VI protein Hes1 is essential for maintenance and proliferation of neural progenitor cells, ensuring temporal control of differentiation competency, whilst also being required for boundary formation, structural integrity and playing later roles in gliogenesis and neuronal protection; comprehensively reviewed in Ref. [4]. Given its pleiotropic roles, it is not surprising that Hes1 is tightly regulated at transcriptional, epigenetic and post-translational levels. In particular, as with regulation described for other bHLH proteins, phosphorylation at individual sites of Hes1 regulates some of its context-dependent effects; for example, JNK1-mediated phosphorylation of S262 in the Hes1 C terminus influences synaptic plasticity in rat cortex [5].

Within the last decade, phosphorylation of tissue-specific class II bHLH proteins has emerged as an important way of restraining differentiation in the context of high cell cycle activity [[6], [7], [8]]. Typically, these multiple phosphorylation events occur on Serine-Proline (SP) or Threonine-Proline (TP) sites in the N and C termini of bHLH proteins, and confer regulation in all three germ layers: multi-site phospho-regulation has been demonstrated for Ngn2, Ascl1, and NeuroD4 in neuroectoderm [6,7,9], MyoD in mesoderm [10] and Neurogenin3 in endoderm [8]. Vertebrate Hes1 homologues of class VI also show a conservation of multiple SP/TP sites across species. Although class VI bHLH proteins are traditionally viewed as transcriptional repressors rather than activators, this raises the intriguing possibility that class VI bHLH proteins may undergo similar multi-site phospho-regulation.

*Xenopus* embryos provide a rapid and accessible *in vivo* model of vertebrate development to study the activity of bHLH transcription factors in differentiation of multiple tissues, for example [[6], [7], [8], [9], [10]]. In particular, the generation of *Xenopus* primary neurons from the neural plate is an established system for probing the interaction between class II proneural bHLH proteins that promote neuronal differentiation, and class VI Hes proteins that inhibit it, for example [11,12].

Here, we use *Xenopus* primary neurogenesis to investigate a role for multi-site phosphorylation in regulation of *Xenopus* Hes1 activity (known as xHes1 or xHairy1). We find that preventing phosphorylation on N-terminal SP/TP sites in xHes1 enhances the ability of xHes1 to inhibit primary neurogenesis driven by three different class II proneural bHLH transcription factors. Mechanistically, we see that preventing phosphorylation of these sites increases stability of xHes1 protein, reduces Ngn2 transcript expression and also leads to greater destabilisation of Ngn2 protein. Furthermore, xHes1 protein is phosphorylated in neural plate stage embryos, and *in vitro* kinase assay sensitivity indicates a potential role for cell-cycle phase dependent regulation.

## 2 Materials and methods

### 2.1 Cloning

Wild-type (WT) *Xenopus* xHes1 and *Xenopus* NeuroD1 were cloned into pCS2 with a single C terminal HA tag. *Xenopus* Ngn2 and mouse Ascl1 have been described previously [6,7]. 5T/S-A xHes1 and 3T/S-A xHes1 were generated by QuikChangeII Site-Directed Mutagenesis Kit (Agilent Technologies). All primers available on request. Nucleotide and protein sequence alignments were conducted using ClustalW [13].

### 2.2 *Xenopus laevis* embryo manipulation

All work has been carried out under UK Home Office Licence and in accordance with the UK Animals (Scientific Procedures) Act, 1986 and associated guidelines. A description of experiments using ARRIVE guidelines is provided in Ref. [14]. Acquisition of *X.laevis* embryos, preparation and injection of synthetic mRNA, and staging of embryos were conducted as described [9,14].

### 2.3 In situ hybridisation (ISH)

ISH was performed using dig-oxigenin-labelled anti-sense probes. Semi-quantitative scoring was conducted for gene expression on the injected side of the embryo relative to the uninjected side; grades were assigned: −3, no expression; −2, marked reduction in expression; −1, mild reduction in expression; 0, no change in expression; +1, increased expression within the neural tube only; +2, additional ectopic expression restricted to the dorsal ectoderm; +3, moderate but patchy ectopic expression spreading over the lateral ectoderm; +4, extensive ectopic expression over the lateral ectoderm in a homogenous pattern.

### 2.4 Quantitative real-time PCR

Whole embryo RNA was extracted, cDNAs prepared and qPCR conducted as described [9,10].

### 2.5 Western blotting

*In vitro* kinase assay was conducted as described [8]. Protein extraction, lambda protein phosphatase treatment and assessment of cytoplasmic and chromatin-bound proteins was conducted as described [9]. All bHLH proteins were detected by a single HA tag and antibodies used according to Ref. [9].

### 2.6 Statistical analysis

For western blotting, experiments were performed in independent duplicate or triplicate with representative results shown; protein quantification was conducted using ImageJ as described [9]. For qPCR data, mRNA expression was normalised to housekeeping gene *EF1α*, and for analysis, mRNA levels in injected categories were calculated relative to stage-matched uninjected controls. Mean values and the standard error of the mean (s.e.m.) were calculated from N independent experiments. Statistical significance was determined using a paired two-tailed Student's t-test with not significant = NS; (p < 0.05) = *; (p < 0.025) = **; (p < 0.0125) = ***. For ISH data, experiments were conducted in independent duplicate or triplicate and the N numbers refer to the range of total numbers of embryos in each injection category.

## 3 Results

### 3.1 xHes1 is phosphorylated *in vitro* and *in vivo*, regulating its ability to inhibit endogenous primary neurogenesis

Supplementary Fig. 1 shows xHes1 is expressed in early neural plate stage embryos at the anterior and lateral borders of the neural plate [blue arrows] and in future neural crest [white arrows], consistent with a previous report [15]. In contrast, Ngn2, a master regulator of primary neurogenesis, is expressed from early neural plate stages in the three bilateral stripes of developing primary neurons [black arrows] and [16]. Thus, xHes1 and Ngn2 show complementary expression patterns at neural plate stage, and the spatial location of xHes1 may contribute to limiting the area of primary neurogenesis.

**Fig. 1:**
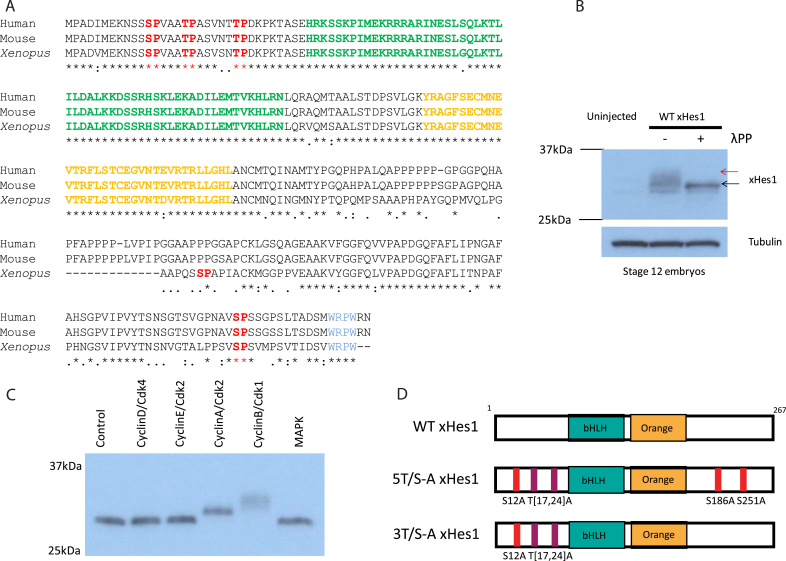
**xHes1 has conserved SP/TP sites and is phosphorylated both *in vivo* and *in vitro*.** (A) Protein sequence alignment for human, mouse and *Xenopus* Hes1. The bHLH domain is shown in green with orange domain in orange and WRPW domain in blue. SP/TP sites are highlighted in red. A consensus line is also shown below the alignment to indicate the degree of conservation at each position: Residues may be identical (*), strongly conserved (:) or weakly conserved (.). (B) Western blot analysis of stage 12 embryos over-expressing 500 pg WT xHes1 mRNA and incubated with or without lambda protein phosphatase. (C) *In vitro* kinase assay showing *in vitro* translated WT xHes1 protein after incubation with recombinant Cyclin/Cdks as labelled. (D) Schematic representation of WT xHes1 protein and phospho-mutant variants, showing approximate location of SP/TP sites that are mutated to AP in each. (For interpretation of the references to colour in this figure legend, the reader is referred to the Web version of this article.)

Sequence alignment of human, mouse and *Xenopus* Hes1 proteins demonstrates strong sequence conservation, particularly in the N terminal half of the protein (Fig. 1A). *Xenopus* xHes1 protein has five serine-proline/threonine-proline (SP/TP) sites, that could potentially be phosphorylated by proline-directed kinases [[6], [7], [8], [9], [10]]. Three of these sites are grouped in the highly conserved N terminus. Of the remaining two sites in the C terminus, one is not conserved with the mouse or human proteins and the other is near to the WRPW domain and has been previously reported as a phosphorylation site enhancing repressor activity in rat cortex [5].

To determine whether xHes1 is phosphorylated *in vivo* in early *Xenopus* development, we expressed Wild-Type (WT) xHes1 in embryos by mRNA injection. Western blotting of protein extracts from stage 12 embryos reveal that WT xHes1 migrates as more than one band (Fig. 1B; red arrow at uppermost band). These forms are reduced to a faster migrating band (black arrow) in the presence of lambda protein phosphatase, indicating that WT xHes1 is indeed phosphorylated *in vivo* on at least one site. To test whether xHes1, in common with multiple tissue-specific bHLH factors [[6], [7], [8]], can be phosphorylated by Cyclin-dependent kinases, we incubated *in vitro* translated WT xHes1 protein with a range of cell-cycle associated Cyclin/Cdk combinations, or with MAPK, another proline directed kinase (Fig. 1C). Significant retardation of migration after separation by SDS-PAGE indicates that WT xHes1 is strongly phosphorylated by CyclinB/Cdk1 on more than one site *in vitro*, and is also phosphorylated to a lesser extent by CyclinA/Cdk2, but not by CyclinD/Cdk4, CyclinE/Cdk2 or MAPK. This suggests that xHes1 may undergo cell-cycle dependent phosphorylation in the G2/M phase of the cell cycle.

**Fig. 2:**
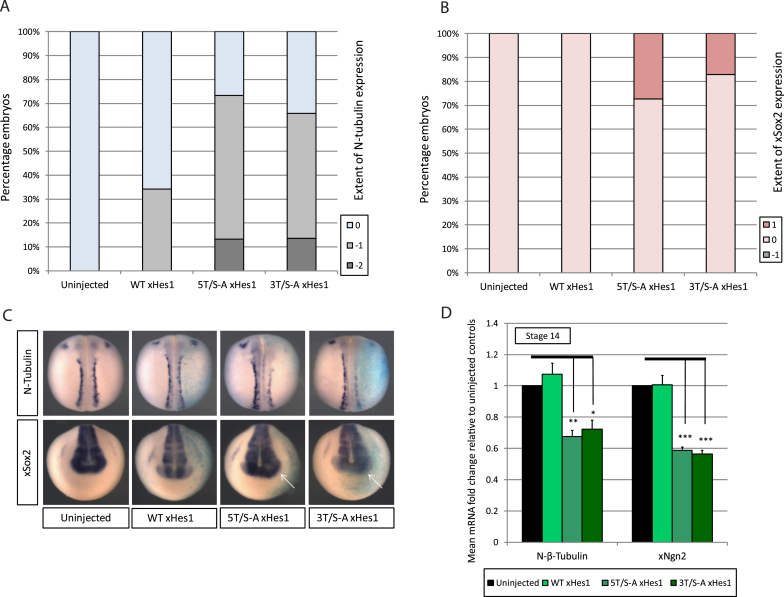
**xHes1 phospho-status regulates inhibition of endogenous primary neurogenesis.** Embryos were injected with 50 pg xHes1 mRNA and assayed at stage 18 by ISH for expression of N-tubulin (A; [N = 30–44]) or xSox2 (B; [N = 22–41]), with representative images shown in (C); N-tubulin dorsal view, xSox2 rostral view. (D) Embryos were assayed by qPCR at stage 14 [N = 3]; significance relative to uninjected embryos (black lines).

xHes1 is known to inhibit primary neurogenesis driven by class II bHLH proneural proteins [12], and this can be used as a measure of xHes1 activity. To explore the effects of manipulating xHes1 phospho-status on inhibition of primary neurogenesis, two phospho-mutant xHes1 constructs were designed (Fig. 1D), either mutating all five potential SP/TP sites (5T/S-A) to alanine-proline (AP), or mutating only the three highly conserved N terminal SP/TP sites (3T/S-A). mRNA encoding each construct was unilaterally injected at the two-cell stage, and embryos were assayed at stage 18 for expression of neural-β-tubulin (N-tubulin) as a marker of differentiated primary neurons (Fig. 2A+C), comparing the injected and uninjected sides of the embryo. At this level of expression, WT xHes1 inhibits endogenous neurogenesis in 30% of embryos. By comparison, both 3T/S-A and 5T/S-A xHes1 are more active, resulting in inhibition of neurogenesis in 70% of embryos.

It has previously been demonstrated that Hes1 can repress Ngn2 activity in progenitor neuroblasts [17]. To determine whether xHes1 can inhibit neuronal differentiation but expand the progenitor population in the neural plate of embryos, expression of early neural progenitor marker xSox2 was investigated (Fig. 2B + C). WT xHes1 injection results in no apparent change in the xSox2 progenitor population, while both 3T/S-A and 5T/S-A xHes1 expand the rostral xSox2 domain [white arrows] in 20–30% of embryos. Thus, preventing phosphorylation of xHes1 protein results in enhanced ability to restrain neuronal differentiation, and instead promoting a progenitor state.

### 3.2 xHes1 phospho-status influences inhibition of primary neurogenesis induced by proneural bHLH proteins

Ngn2 drives primary neurogenesis in *Xenopus* [16], while Hes1 can repress transcription of Ngn2 in mammalian cells [17]. We next compared the effects of WT and phospho-mutant xHes1 on Ngn2 expression in early neural plate stage *Xenopus* embryos, as measured by qPCR (Fig. 2D). At this developmental stage, WT xHes1 has no effect on Ngn2 expression but 3T/S-A and 5T/S-A xHes1 both reduce expression of Ngn2 and N-tubulin by 30–40%, again indicating enhanced activity of the phospho-mutants relative to WT xHes1. Similar repressive activity of 3T/S-A and 5T/S-A xHes1 indicates that the most important phospho-regulatory sites may reside in the N-terminus of xHes1, as these are mutated in both proteins. Hence, we concentrated on defining the function of the minimally mutated 3T/S-A xHes1 in comparison to the WT protein.

**Fig. 3:**
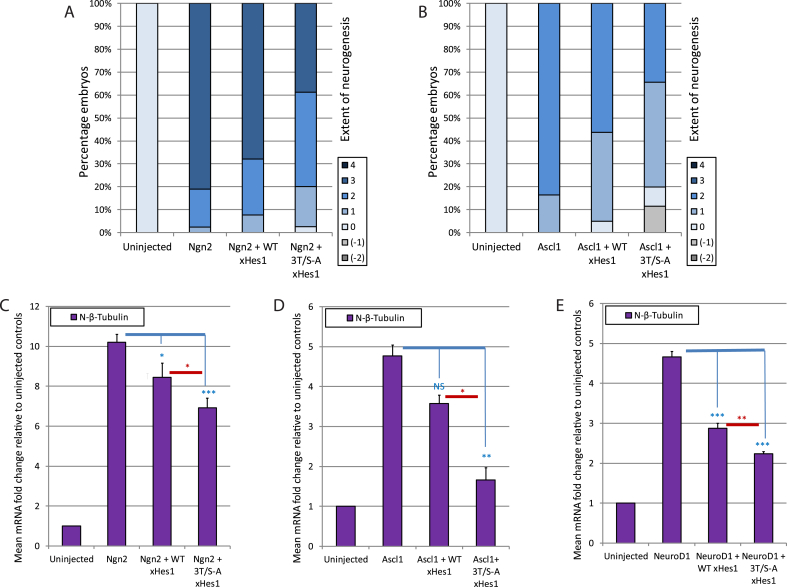
**xHes1 inhibits ectopic neurogenesis induced by three different proneural proteins.** Embryos were co-injected with mRNA encoding either WT or 3T/S-A xHes1 in the presence of one of three different proneural proteins. ISH scores are shown for Ngn2 (A; [N = 78–84]) and Ascl1 (B; [N = 96–104]). qPCR data is shown for Ngn2 (C; [N = 4]), Ascl1 (D; [N = 3]) and NeuroD1 (E; [N = 4]). See methods for grading system.

xHes1 has previously been shown to inhibit the activity of over-expressed Ngn2 in *Xenopus* embryos [12], suggesting an additional level of regulation downstream of Ngn2 transcription. We next sought to investigate whether xHes1 phosphorylation influences this regulation and WT or 3T/S-A xHes1 mRNAs were co-injected with mRNA encoding Ngn2. Embryos were assayed at stage 18 by ISH and qPCR for expression of N-tubulin (Fig. 3A+C).

Ngn2 induces ectopic N-tubulin expression outside the neural tube in 98% of embryos; in 17% this ectopic expression is restricted to the dorsal ectoderm (grade 2) but in 81% this occurs more widely across the lateral ectoderm (grade 3). WT xHes1 co-expression results in a small reduction in the extent of ectopic neurogenesis, while 3T/S-A xHes1 shows enhanced ability to limit ectopic neurogenesis to the neural tube and dorsal ectoderm.

Ascl1 is another class II bHLH transcription factor particularly associated with GABAergic and noradrenergic neurogenesis in *Xenopus* embryos [18,19], while NeuroD1 is a proneural protein acting downstream of Ngn2 in the primary neurogenesis cascade. All three factors are able to induce ectopic primary neurons when over-expressed [7,12]. As with Ngn2, the inhibitory activity of WT and 3T/S-A xHes1 was tested with co-injected Ascl1 or NeuroD1 mRNA (Fig. 3B, D, E). Relative to WT xHes1, preventing N terminal phosphorylation in 3T/S-A xHes1 enhances the inhibitory effects of the xHes1 protein and limits ectopic primary neurogenesis induced by all three proneural transcription factors. Additionally, as NeuroD1 is a direct transcriptional target of Ngn2 [20], the proneural activity of induced NeuroD1 may contribute to the phenotype seen on over-expression of Ngn2. The ability of xHes1 to restrict ectopic neurogenesis induced by both NeuroD1 and Ngn2 may therefore contribute to the inhibitory effects at multiple levels of the Ngn2-induced primary neuron cascade.

### 3.3 Phosphorylation of xHes1 regulates protein stability

**Fig. 4:**
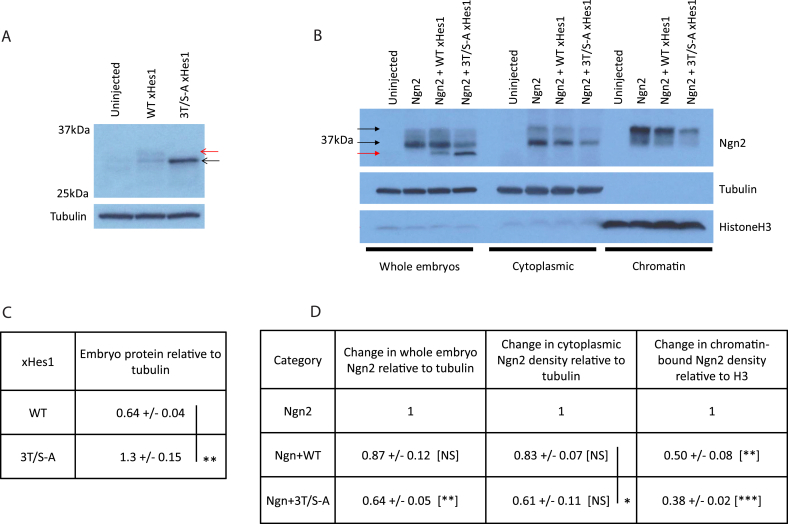
**Phospho-mutant xHes1 has enhanced protein stability relative to WT and reduces both total Ngn2 and chromatin-bound Ngn2 protein.** (A) Western blot analysis of stage 11 embryos over-expressing 500 pg xHes1 mRNA and with xHes1 protein density calculated relative to tubulin in (C) [N = 3]. (B) Western blot analysis of whole embryo extracts from embryos overexpressing 150 pg Ngn2 and 500 pg xHes1 mRNA, and cytoplasmic and chromatin fractions from cross-linked stage 13 embryos injected with 250 pg mRNA of Ngn2 and xHes1; relative protein quantification in (D) [N = 3]; statistics by paired students T-test as described in methods.

Preventing phosphorylation of class II bHLH proteins has previously been shown to enhance protein stability [[8], [9], [10]]. When we directly compare migration of WT and 3T/S-A xHes1 proteins after over-expression in stage 11 embryos (Fig. 4A+C), we see that WT xHes1 protein migrates as multiple bands [red arrow], while 3T/S-A xHes1 migrates faster as a single band, confirming that phosphorylation occurs on N-terminal SP/TP sites. When quantitated relative to the tubulin loading controls, phospho-mutant 3T/S-A xHes1 protein accumulates to twice the level of the combined phospho-forms of the WT. Thus, the WT protein can be destabilised by phosphorylation of these N terminal SP/TP sites and the enhanced stability of the phospho-mutant xHes1 may contribute to its superior ability to inhibit Ngn2 expression and neurogenesis.

### 3.4 xHes1 phosphorylation status influences Ngn2 protein stability

In addition to regulation at a gene expression level, many class II proneural transcription factors including Ngn2 have been demonstrated to have short half-lives that can be modulated by post-translational modification including phosphorylation [21,22]. Stability is also controlled by protein-protein interactions, including between class II proneural proteins and their heterodimeric class I E protein partners [21], and dimerisation is required for DNA binding [23]. As Hes1 can sequester E proteins from class II proneural transcription factors [24], we next investigated the effect of WT and 3T/S-A xHes1 on the stability and chromatin binding of Ngn2 in embryos.

After co-injecting mRNAs encoding WT or 3T/S-A xHes1 along with Ngn2, we determined protein expression levels in stage 11 whole embryo extracts (Fig. 4B; Ngn2, black arrows; xHes1 red arrow). A lower dose of xHes1 mRNA was required to improve embryo survival for chromatin analysis, where embryos were cross linked at stage 13 prior to extraction of cytoplasmic and chromatin fractions (Fig. 4B). When Ngn2 is quantified relative to tubulin in whole embryo extracts (Fig. 4D), Ngn2 protein is reduced to 87% of the control level in the presence of WT xHes1, but shows a more pronounced reduction to 64% of the control level when phospho-mutant xHes1 is co-injected. Additionally, when embryos are fractionated, Ngn2 protein in cytoplasmic extracts shows a corresponding reduction in the presence of WT and 3T/S-A xHes1 that is quantitatively similar to that described for the whole embryo extracts. Thus, in addition to a greater reduction in Ngn2 at transcript level (Fig. 2D), we also see that 3T/S-A xHes1 may be inhibiting Ngn2 via a reduction in Ngn2 protein stability. If xHes1 is destabilising Ngn2 by sequestering E protein partners, one might expect a greater effect on stability of chromatin-bound Ngn2 that is dependent on dimerisation [23], compared to protein in the cytoplasm. Indeed, chromatin-bound Ngn2 is reduced to 50% and 38% of control levels in the presence of WT and 3T/S-A xHes1 respectively; a greater reduction than the corresponding 83% and 61% decreases in cytoplasmic Ngn2 protein.

## 4 Discussion

Using a *Xenopus* model of neurogenesis, here we report that Hes1 homologue xHes1 is phosphorylated *in vivo* on highly conserved SP/TP residues in the N terminus of the protein. Preventing modification of these sites increases xHes1 protein stability and enhances its inhibitory effects on both endogenous and ectopic proneural protein induced neurogenesis, indicating that class VI bHLH proteins can be regulated by SP/TP phosphorylation in an analogous way to that previously described for class II proneural bHLH factors [[6], [7], [8], [9], [10]].

Furthermore, we provide an insight in to the multiple levels of interaction between xHes1 and Ngn2 during primary neurogenesis. xHes1 reduces Ngn2 transcript expression and additionally has post translational effects to destabilise the Ngn2 protein and inhibit proneural activity of downstream factor NeuroD1. Mechanistically, Hes1 has previously been described as a transcriptional repressor with multiple modes of action. For example, direct repression of proneural gene expression via recruitment of the co-repressor Groucho/TLE to N-boxes in the promoter regions of proneural genes [25]; Hes1 binding of E protein partners, titrating them away from proneural bHLHs so reducing their activity [24]; competition between Hes1 homodimers and proneural/E protein heterodimers at certain targets [5]; DNA-independent protein tethering to recruit repressor proteins to target genes bound by proneural/E protein heterodimers [25]; or a mutual degradation mode in *Drosophila* with a tripartite complex of Hes/proneural/E protein components [26]. Preventing phosphorylation of xHes1 enhances its stability, and is consistent with increasing its repressive activity via all but this last mutual degradation mechanism. Using whole embryo bulk chromatin methods, we have been unable to detect chromatin-bound xHes1, possibly due to the highly unstable properties of the xHes1 protein. Nevertheless, ectopic 3T/S-A xHes1 is particularly effective at reducing both the total amount of Ngn2 protein and the chromatin-bound Ngn2 fraction (Fig. 4). This may support a prominent role for E protein sequestration in the mechanism of action of the phospho-mutant xHes1; an effect that would influence both the stability and chromatin binding of the Ngn2 protein [21,23].

How might Cdk-dependent phosphorylation of xHes1 and proneural proteins function in co-ordination to control neurogenesis? Interestingly, Ngn2 and Ascl1 are reported to be most sensitive to phosphorylation by Cdk2 [6,7], whereas here we find that xHes1 is highly sensitive to Cdk1, suggesting that cell-cycle phase-dependent phosphorylation may further fine-tune the relative activities of these two classes of proteins. Phosphorylation of xHes1 at G2/M phase and resultant protein destabilisation is also reminiscent of that described for MyoD to ensure release from chromosomes and allowing mitotic progression [27]. Similarly, cdc2 (Cdk1) mediated phosphorylation of Hes1 co-repressor Groucho occurs in G2/M to reduce chromatin association and repressor activity [28]. Thus, Cdk1-mediated phosphorylation of xHes1 may augment this relief of transcriptional repression during mitosis. Furthermore, in mammalian cells, the onset of differentiation is assumed to occur in the G1 phase but with the decision to differentiate may be in the preceding cell cycle [29]. As such, the phosphorylation of xHes1 in the late G2/M phase may relieve inhibition of class II proneurals to allow the transition to differentiation in the next G1.

It is clear that the interaction between class VI Hes proteins and class II proneural proteins is complex; Ascl1, Ngn2 and Hes1 display dynamic oscillatory antagonism in neuronal progenitors, while a transition to sustained expression (Ascl1/Ngn2) or repression (Hes1) accompanies the switch to neuronal differentiation [30]. It is likely that regulation by multi-site phosphorylation of both class II and class IV bHLH factors acts alongside other regulatory mechanisms (reviewed in Ref. [31]), contributing to this exquisite control and its co-ordination with the cell cycle.

## Figures and Tables

**Supplementary material:**
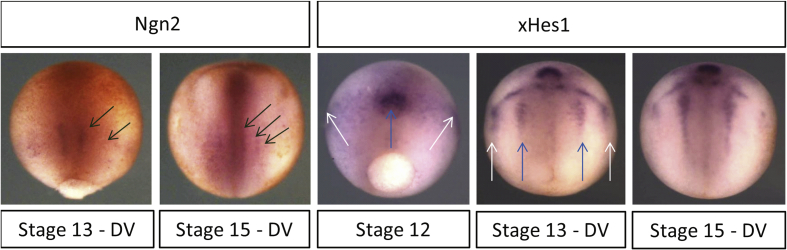
:Supplementary Figure 1: Endogenous expression of xHes1 and Ngn2.Endogenous expression of Ngn2 and xHes1 in stage 13 and 15 embryos revealed by ISH; DV, dorso-ventral view.
